# Non-invasive quantitative measures of qualitative grading effectiveness as the indices of acute radiation dermatitis in breast cancer patients

**DOI:** 10.1007/s12282-020-01082-3

**Published:** 2020-05-03

**Authors:** Hiroshi Sekine, Yoshikazu Kijima, Masao Kobayashi, Jun Itami, Kana Takahashi, Hiroshi Igaki, Yasuo Nakai, Hitoshi Mizutani, Yoshihito Nomoto, Katsuko Kikuchi, Haruo Matsushita, Keiko Nozawa

**Affiliations:** 1 Department of Radiology, The Jikei University School of Medicine, The Jikei University Daisan Hospital, 4-11-1 Izumi-Honcyo, Komae, Tokyo, 201-8601 Japan; 2 Department of Radiation Oncology, National Cancer Center Hospital, Tokyo, Japan; 3 Department of Dermatology, Mie University Graduate School of Medicine, Tsu, Mie Japan; 4 Sakuragi Memorial Hospital, Matsusaka, Mie Japan; 5 Department of Radiation Oncology, Mie University Graduate School of Medicine, Tsu, Mie Japan; 6 Sendai Taihaku Dermatology Clinic, Sendai, Miyagi Japan; 7 Department of Dermatology, Tohoku University Graduate School of Medicine, Sendai, Miyagi Japan; 8 Department of Radiation Oncology, Tohoku University Graduate School of Medicine, Sendai, Miyagi Japan; 9 Appearance Support Center, National Cancer Center Hospital, Tokyo, Japan

**Keywords:** Radiation dermatitis, Erythema, Pigmentation, Skin water content, Transepidermal water loss

## Abstract

**Background:**

Recent improvement of machinery evaluation for the skin changes in various therapies enabled us to evaluate fine changes quantitatively. In this study, we performed evaluation of the changes in radiation dermatitis (RD) using quantitative and qualitative methods, and verified the validity of the conventional qualitative assessment for clinical use.

**Methods:**

Forty-three breast cancer patients received conventional fractionated radiotherapy to whole breast after breast-conserving surgery. Erythema, pigmentation and skin dryness were evaluated qualitatively, and biophysical parameters of RD were measured using a Multi-Display Device MDD4 with a Corneometer for capacitance, a Tewameter for transepidermal water loss (TEWL), a Mexameter for erythema index and melanin index. Measurements were performed periodically until 1 year.

**Results:**

The quantitative manifestations developed serially from skin erythema followed by dryness and pigmentation. Quantitative measurements detected the effects of irradiation earlier than that of qualitative indices. However, the grades of the domains in RD by qualitative and quantitative assessment showed similar time courses and peak periods. However, no significant correlation was observed between the skin dryness grade and skin barrier function. In contrast to serial increase in pigmentation grades, melanin index showed initial decrease followed by marked increase with significant correlation with pigmentation grades.

**Conclusion:**

Subjectively and objectively measured results of RD were almost similar course and peak points through the study. Therefore, validity of the conventional qualitative scoring for RD is confirmed by the present quantitative assessments. Instrumental evaluations revealed the presence of modest inflammatory changes before radiotherapy and long-lasting skin dryness, suggesting indication of intervention for RD.

## Introduction

The skin reactions and effects induced by radiation depend on several factors, including the irradiation area (target volume), fractionation dose, number of fractions, and total radiation time. These skin reactions are known as radiation dermatitis (RD), which includes skin erythema [1, 2], elevated skin temperature [3], skin dryness [4], disappearance of perspiration [5], and increased blood flow in the irradiation field [6, 7]. Biophysical quantitative measurements of these changes could enable the accurate evaluation of various prevention methods and therapies [8–10].

In recent years, high-dose irradiation to the skin has been replaced by adaptive high-precision radiation therapy. However, the quantification of radiation-induced reactions and side effects in normal tissues is insufficient compared to the quantification of the effects of radiation on the tumor. During breast cancer radiotherapy, a high-dose exposure to the skin covering the mammary tissue is unavoidable. The patients having radiotherapy just after breast-conserving surgery (BCS) may experience physical and mental stress due to a severe skin reaction [10–12]. The conventional assessments for skin reactions in radiotherapy are qualitative evaluations with visual inspection or palpation. The most widely used qualitative grading scales are (1) the National Cancer Institute Common Terminology Criteria for Adverse Events (CTCAE) version 4.0 for the classification of acute radiation dermatitis, (2) the Radiation Therapy Oncology Group (RTOG)/European Organization for Research and Treatment of Cancer (EORTC) scale, or (3) the Late Effects Normal Tissue Task Force/Subjective, Objective, Management, and Analysis (LENT/SOMA) scale for the classification of chronic dermatitis [13–15]. Both the CTCAE and RTOG/EORTC scale assess acute radiation effects on a scale from 0 to 4 in increments of 1. Because of limited sensitivity of grading with large increments, several scales with smaller increments (e.g., 0.5) have been developed [16, 17]. Although these scales enable finer classification and grading of radiation-induced skin toxicities, their reliability and validity remain largely unsupported by data because these increments require not only linear regression but also accurate observations. These evaluations have served as the basis for various recommendations regarding the timing and frequency of skin cooling and/or topical corticosteroid application as a method of reducing skin reactions [8–10]. However, few studies have evaluated these skin reactions quantitatively, and it is doubtful whether the results of earlier studies can be replicated scientifically.

This study aimed to use instrumental quantitative measurements to determine the validity of qualitative scales for skin reactions associated with radiotherapy, and to verify the potential association between qualitative and quantitative assessments by analyzing the time-dose effect during whole-breast irradiation.

## Patients and methods

### Participants

This multi-institutional prospective study was performed at the National Cancer Center Hospital, the Jikei University Hospital, Mie University Hospital, and Tohoku University Hospital. Female Japanese patients with newly diagnosed unilateral breast cancer and an Eastern Cooperative Oncology Group performance status of 0 or 1 were enrolled from October 2014 to February 2016 after written informed consents.

All patients underwent BCS followed by whole-breast irradiation. The exclusion criteria for this study were an age > 70 years; previous history of contralateral breast cancer; receipt of boost irradiation after whole-breast irradiation; receipt of chemotherapy before, simultaneously with, or after the completion of irradiation; connective tissue disease; and expected long-term interruption of radiation therapy.

### Radiotherapy

For patients with breast cancer who underwent BCS, postoperative whole-breast radiotherapy was planned using a computed tomography-based, 3-dimensional (3D) radiation therapy planning system. The clinical target volume (CTV) for radiotherapy was defined as the ipsilateral breast. The planning target volume (PTV) was equal to the CTV.

A total dose of 50 Gy in 25 fractions over 5 weeks was delivered to the whole breast via 4- or 6-MV photon beams. Usually, opposed tangential beams with wedge filters or a field-in-field technique were used to avoid administering more than 107% of the prescription dose to the PTV.

### Patient evaluation

Qualitative and quantitative evaluations of RD were conducted using clinician-evaluated grading criteria before the start of radiotherapy (i.e., baseline), once weekly during radiotherapy, and 2 weeks (week 7), 6 weeks (week 11), 3 months (week 17), 6 months (week 26), and 1 year (week 52) after radiotherapy. Objective measurements of skin biophysical parameters were obtained for the qualitative evaluation.

### Qualitative evaluation and quantitative measurements for the assessment of acute RD

Acute RD was graded comprehensively using the CTCAE criteria. RD includes several symptoms in three domains: (1) skin color (erythema and pigmentation); (2) skin characteristics (dryness, roughness, swelling, hardening, and capillary dilatation); and (3) skin sensation (pain, itchy, skin irritation, burning and swelling). These non-subjective symptoms were observed in the acute phase and were evaluated by visual inspection and/or palpation. In this study, the severity of each symptom was evaluated qualitatively and in consensus using a 5-point scale (no change [0], minimum [1], mild [2], moderate [3], and severe [4]). Each patient was evaluated by an experienced radiation oncologist who was blinded to the results of the skin biophysical measurements.

Quantitative measurements were performed after acclimation to an environment of 22–24 °C and relative humidity of 45–60% for 15 min. Two points of measurement on the breast were set at least 5 cm distal from the surgical wounds. Two symmetrically located points were set on the contralateral non-irradiated breast. All measurements were performed by the same investigator. Patients were not permitted to use topical products during the course of radiotherapy unless they complained of severe pain and/or itching. In such cases, the application of non-corticosteroid topical products was not permitted within 4 h prior to radiotherapy and measurements.

The quantitative measures included skin temperature, erythema, pigmentation, and the parameters related to skin barrier function: transepidermal water loss (TEWL) and the skin surface moisture level (capacitance). These biophysical parameters were selected because they were expected to change in response to RD, as noted in previous studies [1–7]. Irradiated and contralateral non-irradiated areas of skin were measured non-invasively using a Multi-Display Device MDD4 (Courage + Khazaka Electronic GmbH, Cologne, Germany) connected with the following probes: A Corneometer to detect the relative water content of the stratum corneum, measured the capacitance of the dielectric medium; skin hydration was measured in relative units on a scale from 0 to 120 [10]. Second, a Tewameter for TEWL was used to measure the water evaporation density gradient from the skin indirectly by the two pairs of sensors (temperature and relative humidity) inside the open style hollow cylinder. The measured values expressed the evaporation rate in g/h/m^2^ [18, 19]. Third, the Mexameter was used for erythema (erythema index) and pigmentation (melanin index) evaluations at 3 wave lengths: 568, 660, and 880 nm [20–22]. The erythema and melanin indices were calculated as follows:

$${\text{Erythema index}} = a_{{\text{E}}} \cdot {\log}\left( {I_{{{66}0{\text{ nm}}}} /I_{{\text{568 nm}}} } \right) + b_{{\text{E}}} ,$$

$${\text{Melanin index}} = a_{{\text{M}}} \cdot {\log}\left( {I_{{{88}0{\text{ nm}}}} /I_{{{66}0{\text{ nm}}}} } \right) + b_{{\text{M}}} ,$$

where *I*_568 nm_, *I*_660 nm_, and *I*_880 nm_ represent the reflectance of each wavelength, *a*_E_ and *b*_E_ are the coefficients for erythema, and *a*_M_ and *b*_M_ are the coefficients for melanin.

The skin surface temperature was measured using a thermometer (THERMO PIPPER^®^, Sato-Shoji, Kawasaki, Japan).

The final objective measurements are described as ratios, which were used to calculate deviations from the simultaneously measured values of the non-irradiated breast. The following formula was used: objective measure in irradiated breast/objective measure in control breast.

### Statistical analysis

For quantitative measurements, the mean value of two data points was used in the analysis. When the clinically evaluated qualitative grades of the two points did not match, the average value was recorded; although the grades were not linear but stepwise, they were compared using rank tests. Measurements of biophysical parameters at baseline versus the indicated time were compared using the signed-rank test. Quantitative measurements corresponding to grade 0 of each clinically evaluated symptom at baseline were compared with the biophysical value corresponding to grade 0 at the indicated time using the signed-rank test. Correlations between clinician-evaluated grading criteria and changes in skin biophysical parameters were determined using Spearman’s correlation test. A correlation coefficient |*r*| of > 0.7, > 0.4–0.7, > 0.2–0.4, and ≤ 0.2 indicated a strong, moderate, or weak correlation or a near-lack of correlation, respectively.

In this study, statistical significance was assumed at a *p* value of ≤ 0.01. Statistical analysis was performed using the built-in functions of Mathematica, version 11.3 (Wolfram Research, Inc., Champaign, IL, USA).

The study was conducted in accordance with the Declaration of Helsinki as well as the Ethical guidelines for medical health research involving human subjects (2014) by Japanese Ministry of Health and Welfare and Ministry of Education, Culture and Technology. This study was conducted with the approval of the Ethics Committee of National Cancer Center Hospital (approved No.: 2014-195).

## Results

A total of 43 patients (median age: 57.5 years, range: 29–71) were included in the initial analysis. Three patients withdrew their consent during and after radiotherapy because of the extra time required for each measurement. Finally, 40 patients were followed until 1 year after irradiation.

### Changes of qualitative grades in RD

**Fig. 1:**
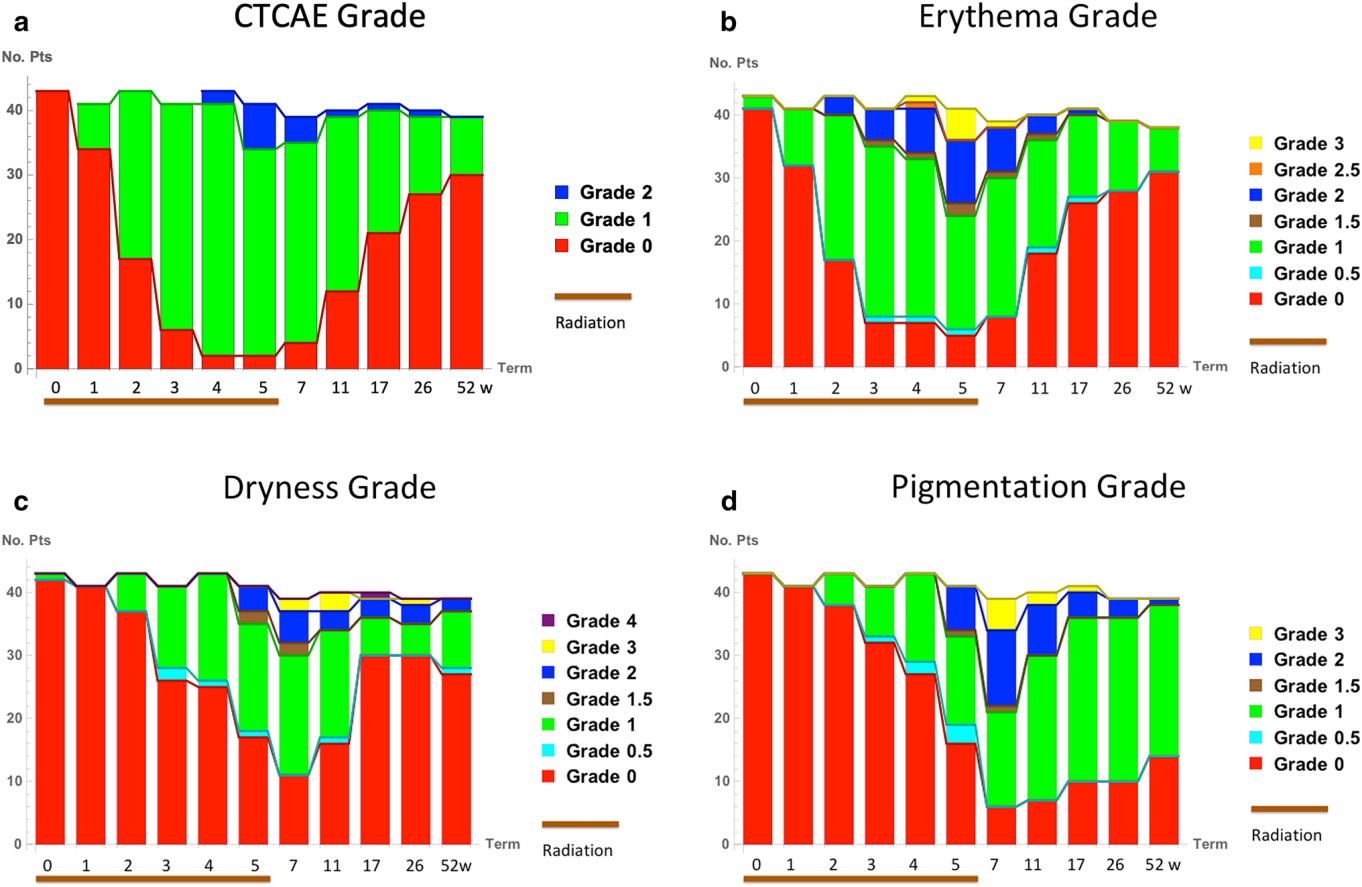
Changes over time in the numbers of patients corresponding to each of the following grades: **a** CTCAE grade, **b** erythema grade, **c** dryness grade, and **d** pigmentation grade. 0: before radiotherapy (baseline); 1, 2, 3, 4, 5: weeks during radiotherapy; 7: 2 weeks after radiotherapy; 11: 6 weeks after radiotherapy; 17: 3 months after radiotherapy; 26: 6 months after radiotherapy; 52: 1 year after radiotherapy. *CTCAE* Common Terminology Criteria for Adverse Events

The majority of patients developed minimum or moderate acute RD (CTCAE grade 1 or 2) (Fig. 1a). CTCAE grade 1 developed in some patients at the 1st week of radiotherapy and grade 2 at week 4. Erythema grade 1 also developed at week 1 and grade 2 at week 2 in some patients (Fig. 1b). Dryness and pigmentation grade 1 developed at week 2 but grade 2 started at week 5 (Fig. 1c, d). The peak reaction time of CTCAE and erythema grade were at the end of radiotherapy: week 5, but those of dryness and pigmentation were two weeks after end of radiotherapy (week 7). When changes in the CTCAE grade were compared with symptom-specific criteria, a moderate correlation was observed between the erythema grade (correlation coefficient = 0.56) (Table 1).

**Table 1:** Correlations of the CTCAE grades with symptom-specific criteria at the peak reaction time of each symptom

	Time	Erythema	Dryness	Pigmentation
CTCAE	Week 5	0.56*		
	Week 7		0.21**	
	Week 7			0.22**

Values are presented as correlation coefficients, determined using Spearman’s correlation test

*CTCAE* Common Terminology Criteria for Adverse Events

*p < 0.01, **Not significant

### Changes of quantitative parameters in RD

**Fig. 2:**
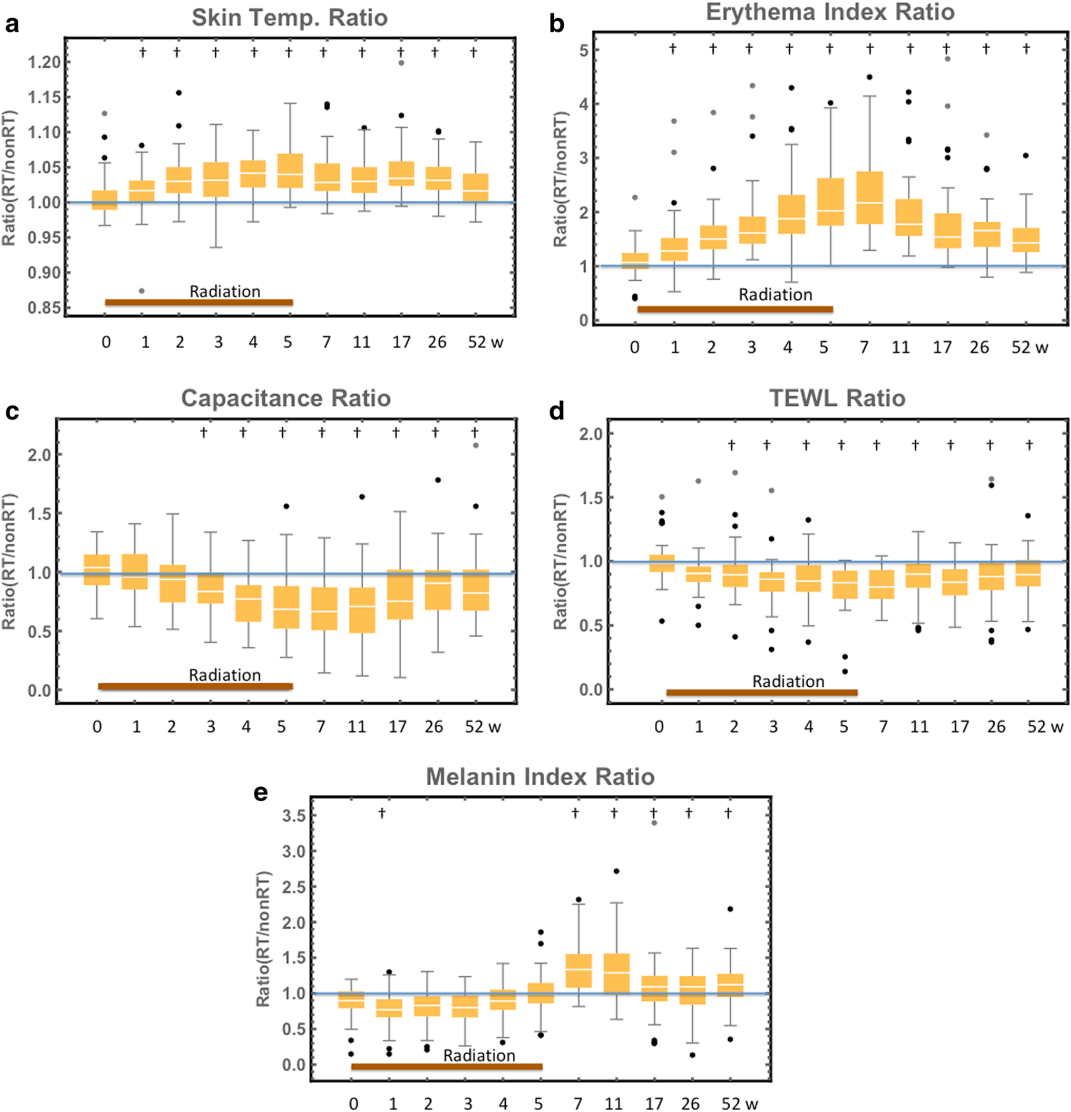
Time course of biophysical parameters: **a** skin temperature ratio, **b** erythema index ratio, **c** capacitance ratio, **d** TEWL ratio, and **e** melanin index ratio. †*p* < 0.01

The skin temperature and erythema index increased significantly from 1st week of irradiation (Fig. 2a, b). However, the TEWL decreased significantly from 2nd week of radiotherapy and capacitance from 3rd week of radiotherapy (Fig. 2c, d). Interestingly, the melanin index ratio at 1 week of irradiation (< 10 Gy) was significantly lower than the baseline value (baseline: 0.886 vs. 1 week of irradiation: 0.769, *p* < 0.01), and remained under 1.0 until end of radiotherapy: week 5 (Fig. 2e). Melanin index elevated significantly after week 7(Fig. 2e). Skin temperature showed the most significant difference in biophysical parameters (peak period) during radiotherapy (week 4); however, other parameters showed maximum difference at 2 weeks after irradiation. Median index ratio for each of the biophysical parameters before radiotherapy along with the maximum difference in time is shown in Table 2. Before radiotherapy, the median values of skin temperature and erythema index ratio are slightly elevated from 1.0 and melanin index ratio was under 1.0. Capacitance also slightly elevated from 1.0 but not TEWL (Fig. 2).

**Table 2:** Changes in quantitative parameters from before radiotherapy to the peak time

	Peak time	Paired number of subjects	Median value		*p* value
			Before radiotherapy*	Peak time**	
Skin temperature	Week 4	43	1.00	1.04	< 0.01
Erythema index	Week 7	38	1.06	2.17	< 0.01
Capacitance	Week 7	39	1.04	0.66	< 0.01
TEWL	Week 7	39	0.98	0.80	< 0.01
Melanin index	Week 1	41	0.88	0.76	< 0.01
Melanin index	Week 7	38	0.91	1.44	< 0.01

*Objective measure in operated breast before radiotherapy/objective measure in control breast before radiotherapy

**Objective measure in irradiated breast at peak period/objective measure in control breast at peak period

### Deviation of quantitative parameters in grade 0 patients through the study

The analysis of CTCAE and erythema grades revealed a clear and significant difference between grade 0 before irradiation and grade 0 at 1 and 2 weeks of irradiation and between 6 weeks after irradiation and 1 year later (*p* < 0.01, Table 3). In addition, as the degree of skin dryness progressed, the corresponding quantitative value judged as qualitative grade 0 decreased significantly (capacitance: 4 weeks of irradiation to 2 weeks following irradiation, TEWL; 2 weeks of irradiation to 2 weeks, 3 months and 1 year following irradiation) (Table 3). There was no significant difference observed in the measured melanin index associated with grade 0 pigmentation at any measurement time except for at 1 week of irradiation (Table 3).

**Table 3:** Quantitative values corresponding to grade 0 at baseline (before irradiation) were compared with the quantitative values classified as grade 0 at each time point (signed-rank test)

Time	CTCAE Grade 0 (Erythema index)				Erythema Grade 0 (Erythema index)				Skin Dryness Grade 0 (Capacitance)				Skin Dryness Grade 0 (TEWL)				Pigmentation Grade 0 (Melanin index)			
	Paired number of Pts	Median (baseline)	Median (index time)	p*	Paired number of Pts	Median (baseline)	Median (index time)	*p*	Paired number of Pts	Median (baseline)	Median (index time)	*p*	Paired number of Pts	Median (baseline)	Median (index time)	*p*	Paired number of Pts	Median (baseline)	Median (index time)	*p*
1	34	1.11	1.27	< 0.01	32	1.13	1.29	< 0.01	40	1.03	0.97	0.71	40	0.98	0.91	0.03	41	0.89	0.77	< 0.01
2	17	1.02	1.43	< 0.01	17	1.02	1.30	< 0.01	37	1.01	0.94	0.12	37	0.98	0.90	< 0.01	38	0.88	0.83	0.07
3	6	1.21	1.66	0.06	7	1.17	1.60	0.03	26	1.02	0.88	0.02	26	0.99	0.88	< 0.01	32	0.83	0.78	0.09
4	2	1.29	1.69	0.37	7	1.15	1.72	0.02	25	1.05	0.78	< 0.01	25	0.98	0.83	< 0.01	27	0.90	0.87	0.39
5	2	1.21	1.37	1.00	5	1.17	1.73	0.11	17	1.08	0.65	< 0.01	17	0.97	0.85	< 0.01	16	0.82	0.90	0.62
6	4	1.32	1.82	0.20	8	1.29	2.07	0.02	11	1.12	0.82	< 0.01	11	1.01	0.80	< 0.01	5	0.99	1.05	0.11
7	12	1.07	2.08	< 0.01	18	1.11	2.08	< 0.01	15	1.11	0.91	0.06	15	0.96	0.97	0.51	7	0.90	1.11	0.03
8	21	1.02	1.54	< 0.01	26	1.04	1.52	< 0.01	29	1.05	0.89	0.04	29	0.97	0.90	< 0.01	10	0.81	0.94	1.00
9	27	1.09	1.51	< 0.01	28	1.06	1.49	< 0.01	28	1.05	0.88	0.01	29	1.00	0.90	0.02	10	0.81	1.06	0.68
10	30	1.06	1.40	< 0.01	31	1.04	1.42	< 0.01	26	1.04	0.83	0.03	26	0.98	0.86	< 0.01	14	0.81	1.04	0.02

Time 1, 2, 3, 4, 5: weeks during radiotherapy, 6: 2 weeks after radiotherapy, 7: 6 weeks after radiotherapy, 8: 3 months after radiotherapy, 9: 6 months after radiotherapy, 10: 1 year after radiotherapy

*CTCAE* Common Terminology Criteria for Adverse Events, *Pts* patients, *p* *p* value, *p < 0.01 indicated statistical significance, *TEWL* transepidermal water loss

### Correlation of qualitative grading with quantitative indices

Interestingly, CTCAE grade showed significant positive relation with erythema index and negative relation with TEWL at the end of radiotherapy (week 5) (Table 4). Skin temperature rapidly showed peak activity as early as week 4 during radiotherapy. CTCAE and erythema grades showed maximum reaction at the end of radiotherapy (week 5). However, all other indices: dryness, pigmentation grades, erythema index, capacitance, TEWL and melanin index showed peak two weeks after radiotherapy (week 7). Erythema grades showed clear relation with erythema index but no other index at the end of radiotherapy (week 5) (Table 4). Although weak correlations between the skin dryness grade, capacitance and TEWL were observed (correlation coefficient = − 0.32 for capacitance and 0.22 for TEWL), the grade of skin dryness was not significantly associated with TEWL (*p* = 0.16) (Table 4). Pigmentation grade showed clear positive relation with melanin index (correlation coefficient = 0.50, *p* = 0.0012) at two weeks after radiotherapy (week 7) (Table 4). The qualitative grading and quantitative indices showed similar peak period and co-relation around the peak period. CTCAE- and erythema-related indices showed co-relation at week 5, and dryness- and pigmentation-related indices showed relation at week 7 (Table 4).

**Table 4:** Correlations of qualitative grades with changes in quantitative parameters at each time with maximum symptoms

	Peak time	Erythema index	Skin temperature	Capacitance	TEWL	Melanin index
CTCAE	Week 5	0.40*	− 0.005	0.27	− 0.32*	0.19
	Week 7			0.08	− 0.1	0.083
	Week 11			0.27		
Erythema	Week 5	0.35*	0.19			
Skin dryness	Week 7			− 0.32*	− 0.22	
Pigmentation	Week 7					0.50**

Values are shown as correlation coefficients, which were determined using Spearman’s correlation test

*CTCAE* Common Terminology Criteria for Adverse Events, *temp* temperature, *TEWL* transepidermal water loss

*p < 0.05, **p < 0.01

### Analysis of qualitative grading in higher qualitative grades

**Fig. 3:**
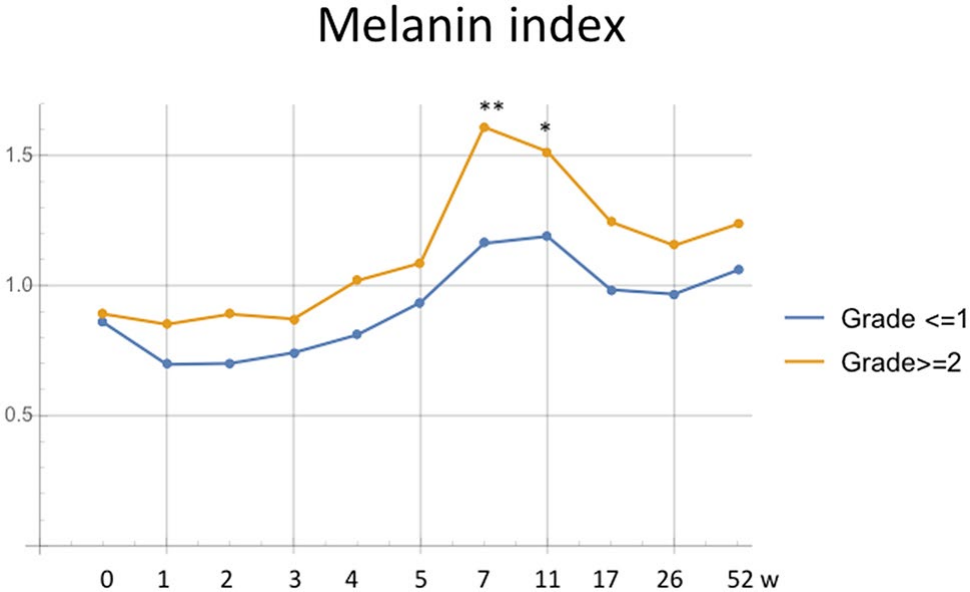
Time course of mean melanin index ratio of patients with pigmentation grade 2 or more and grade 1 or less. **p < 0.001, *p < 0.05

Even CTCAE grade limited grade 2 in this study, grade 3 cases developed in erythema, dryness and pigmentation grades. To elucidate factors effects to severe RD, we analyzed quantitative data between　qualitative grade 2 and higher groups and others. Erythema grades in CTCAE grade 2 group showed higher level through the study without significance (data not shown). Erythema index at grade 2 and higher group showed higher levels through radiotherapy and 2 weeks after radiotherapy without significance. Skin temperature, capacitance and TEWL showed no difference on both groups. However, melanin index in pigmentation grade 2 and more group showed higher levels through the study and showed significant difference at 6 and 12 weeks after radiotherapy (Fig. 3).

## Discussion

Our findings indicate that biophysical quantitative measurements could detect the fine effects of irradiation even at a very early stage with unexpected paradoxical results for pigmentation. The quantitative evaluation sensitively detected modest elevation of skin temperature, erythema index and decrease in melanin index compared with the control breast before radiotherapies. This suggests the presence of mild inflammation due to surgical therapies before radiotherapy, which is undetectable by qualitative evaluation. Notably, skin temperature significantly elevated at the first week of radiotherapy and showed the peak earliest among quantitative indices at week 4 of radiotherapy. Skin temperature is possibly the most sensitive index for acute RD. Interestingly, both the qualitative grades of erythema and erythema index rapidly respond to radiotherapy and showed clear correlation at the end of radiotherapy. Both are expected for good indices for RD at early phase. The CTCAE grading criteria have been widely used for evaluation of RD; however, quantitative analysis of CTCAE grading is limited [23]. The CTCAE grading represents sum of several symptoms in RD, but suitability of CTCAE for acute or late phase of RD was unclear. Among qualitative 4 grades, CTCAE showed a moderate correlation erythema grade at the end of radiotherapy week 5, which indicates both are useful for indices for early phase RD (Table 1). Absence of pigmentation domain in CTCAE grading is also supportive for this result.

The qualitative skin dryness and pigmentation grades as well as the quantitative capacitance, TEWL and pigmentation became obvious 2 or 3 weeks after early phase indices and lasted until end of the study. Capacitance and TEWL are mainly due to the structural changes of epidermis, which require an elapsed time of 2–3 weeks [4]. Interestingly, all these indices similarly showed their peak at 2 weeks after radiotherapy (week 7). Capacitance and TEWL represent the functional changes in RD, and similarly decreased during radiotherapy with gradual recovery. Capacitance presenting skin surface water content showed exact relation with skin dryness at week 7 as expected. Interestingly, TEWL presenting skin barrier function showed significant relation with CTCAE at the end of radiotherapy (week 5). This supports reliability of CTCAE grading in part for RD from both of appearance and function.

Surprisingly, the melanin index started lower than control breast levels and decreased significantly within the first week of irradiation. It gradually returned to the control breast level at the end of radiotherapy (week 5) and showed significant elevation 2 weeks after radiotherapy. The underlying pathogenesis is unknown, although the influence of an early strong erythema reaction is suspected. For example, a positive correlation was observed between the erythema index and the reflex dose of 660 nm, while a negative correlation was observed between the melanin index and this reflex dose. Thus, an increase in the reflex dose of 660 nm due to strong erythema in the first week may result in a relative decrease in the melanin index. Changes before radiotherapy are also explained similarly and the presence of mild inflammatory reaction by surgical therapy is suspected. In contrast, quantitative pigmentation grade linearly increased from week 2 and developed to grade 2 at the end of radiotherapy. Two weeks after radiotherapy, it peaked with Grade 3 cases and persisted at high levels to the end of the study. This may indicate underestimation of melanin index by machinery measurement.

The determination of correlations between qualitative and quantitative values was an important aim of this study. Here, we observed a moderate correlation of the CTCAE grade with the erythema index ratio at 5 weeks of irradiation, and a weak correlation of the erythema grade with the erythema index ratio at the same time point (Table 4). The erythema grades were distributed widely from grade 0 to grade 3 when compared to the CTCAE grade, and the distribution of the measured erythema index values appeared to be broad, which led to a decrease in the correlation strength. This outcome may be attributable to limitations in the subjective judgment of symptoms.

The changes in quantitative measurements corresponding to a subjective grade of 0 are important from the view point of the reproducibility of the qualitative evaluation over time. Within CTCAE grade 0, the erythema index increased and varied significantly until week 2. Many cases shifted to higher grade after week 3, but this validation was observed from 2 weeks after radiotherapy to end of the study. As we reported recently, instrumental evaluation is advantageous because it enables the sensitive detection of changes in subclinical erythema [24].

Fortunately, no case presented CTCAE grade 3 in this study. Despite the appearance of grade 3 erythema, dryness, and pigmentation, the successful completion of the irradiation without interruption is an indication of the safety and reliability of this regimen. Subjects with high CTCAE, erythema and dryness grades showed no significant difference in skin temperature, erythema index, capacitance and TEWL between subjects with lower grades. Melanin index showed significant higher levels in Grade 2 and more groups than lower group after radiotherapy.

In conclusion, this study revealed the successful detection of time course-dependent skin changes associated with RD of the breast following irradiation for breast cancer. Our results suggest that both quantitative and qualitative methods are indicated for the evaluation of RD.

The purpose of this study was to quantitatively verify the validity of the quantitative evaluation method for acute RD. Instrumental evaluation is somewhat advantageous since it can be applied early with higher sensitivity to assess persistent changes that are undetectable by a visual assessment and palpation. Accordingly, our study presents evidence of the clinical usefulness of both qualitative and quantitative evaluations of skin changes during radiotherapy. In addition, this study revealed the presence of inflammation at the first week of radiation therapy and long-lasting skin dryness and hyperpigmentation, which suggests indication of early intervention and long-term topical therapies for RD.

A larger-scale investigation is warranted, given the relatively small number of subjects in this study.
